# Comparison of the sputum microbiome between patients with stable nontuberculous mycobacterial pulmonary disease and patients requiring treatment

**DOI:** 10.1186/s12866-024-03308-2

**Published:** 2024-05-18

**Authors:** Min Jong Song, Dae Hun Kim, Su-Young Kim, Noeul Kang, Byung Woo Jhun

**Affiliations:** 1 Division of Pulmonary and Critical Care Medicine, Department of Medicine, Samsung Medical Center, Sungkyunkwan University School of Medicine, 81 Irwon-Ro, Gangnam-Gu, Seoul, 06351 Republic of Korea; 2 Division of Allergy, Department of Medicine, Samsung Medical Center, Sungkyunkwan University School of Medicine, Seoul, South Korea

**Keywords:** Nontuberculous mycobacteria, Microbiota, Antibiotics, Diversity, Outcome

## Abstract

**Background:**

We evaluated whether the sputum bacterial microbiome differs between nontuberculous mycobacteria pulmonary disease (NTM-PD) patients with stable disease not requiring antibiotic treatment and those requiring antibiotics.

**Methods:**

We collected sputum samples from 21 clinically stable NTM-PD patients (stable group) and 14 NTM-PD patients needing antibiotic treatment (treatment group). We also obtained 13 follow-up samples from the stable group. We analyzed the 48 samples using 16S rRNA gene sequencing (V3–V4 region) and compared the groups.

**Results:**

In the linear discriminant analysis effect size (LEfSe) analysis, the species *Porphyromonas pasteri*, *Haemophilus parahaemolyticus*, *Prevotella nanceiensis*, and *Gemella haemolysans* were significantly more prevalent in the sputum of the stable group compared to the treatment group. No taxa showed significant differences in alpha-/beta-diversity or LEfSe between the 21 baseline and 13 follow-up sputum samples in the stable group. In the stable group, the genus *Bergeyella* and species *Prevotella oris* were less common in patients who achieved spontaneous culture conversion (*n* = 9) compared to those with persistent NTM positivity (*n* = 12) (effect size 3.04, *p* = 0.039 for *Bergeyella*; effect size 3.64, *p* = 0.033 for *P*. *oris*). In the treatment group, *H*. *parainfluenzae* was more common in patients with treatment success (*n* = 7) than in treatment-refractory patients (*n* = 7) (effect size 4.74, *p* = 0.013).

**Conclusions:**

Our study identified distinct bacterial taxa in the sputum of NTM-PD patients based on disease status. These results suggest the presence of a microbial environment that helps maintain disease stability.

**Supplementary Information:**

The online version contains supplementary material available at 10.1186/s12866-024-03308-2.

## Background

Nontuberculous mycobacteria (NTM) are ubiquitous organisms found in the environment and which can cause pulmonary disease (PD), with the global burden of NTM-PD on the rise [1]. Among the approximately 200 NTM species, *Mycobacterium avium* complex is the most common causative agent of NTM-PD, while *Mycobacterium abscessus* is highly treatment-refractory, making it a growing focus of medical attention [2, 3]. NTM-PD can occur in immunocompromised individuals; however, a significant proportion of NTM-PD patients show no clear evidence of immunodeficiency and have a typical phenotype known as Lady Windermere syndrome [4]. NTM-PD is currently recognized as a multifactorial disease, and it is postulated that the virulence of the NTM species, the host immune system, and the microbial environment interact to play a role in its pathogenesis.

NTM-PD is unique in having a heterogeneous disease course, compared to other respiratory infections. Some NTM-PD patients remain stable without antibiotic therapy, and approximately 30% of NTM-PD patients achieve spontaneous culture conversion [5]. The remaining patients experience clinical and radiological deterioration during follow-up, eventually requiring treatment. Cohort studies have attempted to analyze predictors of the progression of NTM-PD, identifying age, low body weight, and disease extent as possible factors [6–8]. However, no single clinical factor that can clearly distinguish disease progression from stable NTM-PD has been identified.

Recently, an association between the respiratory tract microbial environment and respiratory disease has been reported [9, 10], and the microbiome has been used to elucidate the progression or pathogenesis of various respiratory diseases. Culture-independent techniques, such as targeted sequencing of the 16S rRNA gene, help to identify bacterial microbes, and these techniques can be applied to characterize the taxa present in a respiratory bacterial microbiome. However, there are limited data on the role of the microbiome in the course of NTM-PD [11, 12]. Therefore, this study compared the bacterial microbiomes in the sputum of NTM-PD patients who remained stable without antibiotics and those who required treatment for disease progression to determine whether there were significant differences in the microbiomes of the two groups and, in particular, to evaluate the characteristics of the respiratory microbial environment in NTM-PD patients with stable clinical status. We believe that our findings will improve our understanding of how the respiratory microbial environment influences the course of NTM-PD.

## Methods

### Study participants

**Fig. 1:**
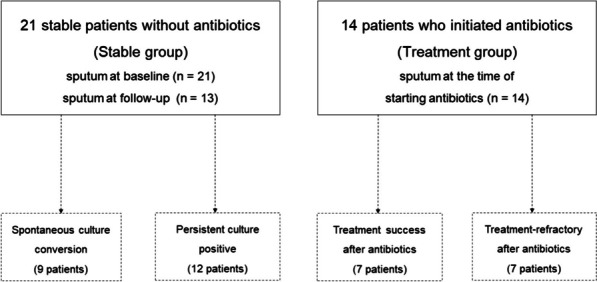
Study patients

From October 2020 to December 2022, we screened patients diagnosed with NTM-PD who had no history of NTM-PD treatment and agreed to provide sputum samples for our microbiome study [12]. All 35 patients who were included in the study had respiratory symptoms, radiological evidence of bronchiectasis or cavity lesions with bronchiolitis, and a positive test for NTM in more than two separate sputum samples (Fig. 1). All patients met the diagnostic criteria outlined in the current guidelines. Because some NTM-PD patients remain stable without antibiotic therapy even after diagnosis, while others experience clinical or radiological deterioration over several months, requiring antibiotic treatment. Thus, the study cohort included 21 stable patients who were under clinical observation without antibiotic treatment (stable group) and 14 patients who had received antibiotic treatment due to clinical and radiological deterioration (treatment group). We collected 34 sputum samples from the stable group, including 21 baseline samples and 13 samples collected more than 6 months later. In the treatment group, we collected 14 samples at the initiation of antibiotic therapy. The 48 sputum samples from the 35 NTM-PD patients were used for bacterial microbiome analysis by 16S rRNA gene sequencing (V3–V4 region). All sputum samples analyzed in our study were collected before antibiotic exposure. The influence of antibiotic exposure was removed in microbiome analysis.

This study was conducted on a subset of individuals from the NTM Registry of Samsung Medical Center (ClinicalTrials.gov: NCT0097080) and was approved by the Institutional Review Board (IRB) of Samsung Medical Center (IRB no. 2008-09-016) [12, 13]. This study is an ongoing research project approved by the IRB to analyze clinical factors and human-derived materials, including sputum and NTM strains, for the investigation of the pathophysiology of NTM-PD patients. The IRB approval has been renewed annually (IRB no. 2008-09-016-041, latest update on November 3, 2023). All study subjects provided written informed consent.

### Sputum specimens

Approximately 3 mL of sputum was collected from each patient. To minimize contamination, the sputum was collected in the morning before any food consumption; for collection, the patients coughed deeply into a container that does not leak. The collected sputum was stored in a deep-freezer at − 80 °C, and the microbiome was analyzed within 3 days.

### DNA extraction from sputum and MiSeq sequencing

DNA extraction from sputum was performed using the MP Biomedicals FastDNA® Spin Kit for soil (MPbio, Santa Ana, CA, USA) according to the manufacturer’s protocol. Then, the concentration and quality of the extracted DNA were measured using the Epoch™ Spectrometer (BioTek, Winooski, VT, USA). DNA concentration was measured with the following parameters: DNA concentration > 15 ng/µl, volume > 20 µl, A260/A280 ratio of 1.8–2.0. The library product was measured using the Bioanalyzer 2100, with these parameters: library product concentration > 12 ng/µl, volume > 20 µl, product size of 400–600 bp. The library product band size was confirmed via agarose gel electrophoresis. In order to analyze the bacterial microbiome in sputum, the V3-V4 region of the 16S rRNA gene in bacteria was amplified.

Based on the MiSeq system protocol for preparing a 16S metagenomics sequencing library, a second PCR (index PCR) was performed to attach an index sequence and an Illumina sequencing adapter to the PCR product using the Nextera XT DNA Library Preparation Kit (Illumina, San Diego, CA, USA). The product of the second PCR was purified using the QIAquick PCR purification kit (Qiagen, Valencia, CA, USA) and quantified using the Quanti-iT PicoGreen dsDNA Assay Kit (Invitrogen, Waltham, MA, USA). The quality of the final library product was measured using the Bioanalyzer 2100 (Agilent, Palo Alto, CA, USA). Samples that did not pass quality control during the DNA extraction and library production steps were excluded from the experiment. The library products that passed quality control were sequenced at CJ Bioscience, Inc. (Seoul, Korea) using the MiSeq Reagent Kit v2 (500-cycles) according to the manufacturer’s instructions and based on the Illumina MiSeq sequencing platform (Illumina).

### Sequence analysis

Microbiome profiling was performed using the 16S-based Microbial Taxonomic Profiling (MTP) platform of the EzBioCloud application, which uses the 16S database version PKSSU.4.030. All raw sequencing data were re-analyzed into individual MTPs using the EzBioCloud pipeline. MTP sets were constructed by grouping these individual MTPs, and comparative analysis between MTP sets was performed after normalization of gene copy numbers since the 16S rRNA gene copy number of bacteria varies by up to 15 times depending on the species. Sequencing reads obtained from 16S rRNA sequence amplification products are classified into species for which the 16S rRNA sequence is known, and the number of reads is used to calculate relative species abundance. The EzBioCloud application we used provides 16S rRNA gene copy number information for over 3,200 species and normalizes the 16S gene copy number for each analyzed bacterial species based on this information.

The relative abundance of sequences was compared between MTP sets using the Wilcoxon rank-sum test. Alpha-diversity was analyzed using the species richness (ACE, Chao1, Jackknife, and number of operational taxonomic units [OTU]), while beta-diversity was evaluated using generalized UniFrac distances and visualized by principal component analysis. Permutational multivariate analysis of variance was used to analyze statistical differences in beta-diversity. To identify differentially distributed taxa between MTP sets, linear discriminant analysis (LDA) was performed to evaluate different groups based on the LDA effect size (LEfSe). LEfSe is a method that uses LDA to assess the effect size of discriminating characteristics between groups. LDA transforms data to maximize group average differences, quantifying the effect size with the LDA score, combining group mean difference and trait variance. Higher LDA scores indicate greater importance in group distinction. We computed LDA scores for each group in LEfSe analysis. Statistical significance was set at *p* < 0.05. Hotelling’s t-test was used to compare bacterial profiles among categories.

We utilized the Phylogenetic Investigation of Communities by Reconstruction of Unobserved States algorithm to estimate the functional profiles of the microbiome identified through 16S rRNA sequencing. The raw sequencing reads underwent computation using the EzBioCloud 16S microbiome pipeline. Functional abundance profiles of the sputum microbiome were annotated through bioinformatics analyses, specifically by multiplying the vector of gene counts for each OTU by the abundance of that OTU in each sample, employing the KEGG (Kyoto Encyclopedia of Genes and Genomes) orthology and pathway database. Predicted profiles were categorized into clusters of KEGG orthology and KEGG pathways, then compared between the stable and treatment groups [14].

## Results

### Study participants

The baseline characteristics of the 35 NTM-PD patients are presented in Table 1. The median age of the patients was 59 years, with the majority being female (91%) and never-smokers (86%). Twenty-three (66%) patients had *M. avium* complex infections. Radiologically, the majority (91%) had the nodular bronchiectatic form of NTM-PD. There were no differences in clinical characteristics between the stable and treatment groups. We additionally compared underlying diseases and blood test results (such as white blood cell, albumin, erythrocyte sedimentation rate, and c-reactive protein) between the stable and treatment groups; however, there were no significant differences between the two groups.

**Table 1:** Clinical characteristics of study patients with NTM-PD

**Characteristics**	**Total (*n* = 35)**	**Stable group (*n* = 21)**	**Treatment group (*n* = 14)**	***P*-value**
Age, years	59.3 (43–78)	61 (45–78)	56 (43–70)	
Female	32 (91)	19 (90)	13 (93)	
Body mass index, kg/m^2^	22.0 (16.8–29)	22.4 (16.8–29.4)	21.2 (18.1–24.4)	
Never smoker	30 (86)	17 (81)	13 (93)	
Underlying disease				
Previous pulmonary tuberculosis	3 (9)	3 (14)	-	0.259
Chronic obstructive pulmonary disease	4 (11)	1 (5)	3 (21)	0.279
Malignancy	5 (14)	4^a^ (19)	1^b^ (7)	0.627
Etiology				
*M. avium* complex	23 (66)	14 (67)	9 (64)	> 0.999
Others	12 (34)	7 (33)	5 (36)	
*M. abscessus*	5/12	5/7	-	
*M. massiliense*	3/12	1/7	2/5	
Mixed infection	4/12	1^c^/7	3^d^/5	
Positive sputum acid-fast bacilli smear	23 (66)	12 (57)	11 (79)	
Laboratory findings at baseline				
White blood cell/μL	5335 (4690–6610)	5535 (4810–6985)	5230 (4330–5730)	0.931
Albumin, g/L	4.4 (4.2–4.5)	4.4 (4.2–4.6)	4.4 (4.2–4.5)	0.494
Erythrocyte sedimentation rate, mm/h	10.5 (6–20)	12.5 (6–22)	9.5 (6–14)	0.891
C-reactive protein, mg/dL	0.06 (0.06–0.12)	0.06 (0.06–0.10)	0.07 (0.05–0.14)	0.889
Radiological form of NTM-PD				
Nodular bronchiectatic form	32 (91)	18 (86)	14 (100)	
Fibrocavitary form	3 (9)	3 (14)	-	
Clinical outcomes				
Spontaneous culture conversion	NA	9 (43)	NA	
Culture conversion after antibiotics	NA	NA	7 (50)	

Data are presented as number (percentage) or median (interquartile range)

*NTM* Nontuberculous mycobacteria, *PD* Pulmonary disease, *NA* Not applicable

^a^Among these four patients, one each had cancer of breast (*n* = 1), cervix (*n* = 1), kidney (*n* = 1) and rectum (*n* = 1)

^b^One patient had lung cancer

^c^One patient had a mixed infection of *M. abscessus/M. intracellulare*

^d^Among these three patients, one each had a mixed infection of *M. avium/M. intracellulare*, *M. abscessus/M. intracellulare*, and *M. abscessus/M. avium*

Twenty-one patients were in the stable group, undergoing observation without antibiotics, while the remaining 14 were in the treatment group due to disease progression requiring antibiotic therapy. Nine patients (43%) in the stable group achieved spontaneous culture conversion without antibiotics, while seven patients (50%) in the treatment group achieved treatment success as determined via culture conversion, defined as three consecutive negative cultures at 4-week intervals, after antibiotic treatment.

### Taxa with differential distributions in sputum of the stable and treatment groups

We performed LEfSe analysis to evaluate differences in microbial distributions between the stable and treatment groups using baseline sputum from both groups (Table 2). At the genus level, *Gemella* was significantly more frequent in the stable group. At the species level, *Porphyromonas pasteri*, *Haemophilus parahaemolyticus*, *Prevotella nanceiensis*, and *Gemella haemolysans* were significantly more abundant in the specimens from the stable group compared to the treatment group (all LDA effect size > 3.00 and *p* < 0.05) (Supplementary Fig. S1).

**Table 2:** LEfSe analysis for bacterial taxa with distribution differences in sputum between the stable and treatment groups

**Taxon name**	**Stable group sputum at baseline (%) (*n* = 21)**	**Treatment group sputum at starting antibiotics (%) (*n* = 14)**	**LDA effect size**	***P*-value**^¶^
Genus				
*Gemella*	0.633	0.28	3.33	0.016
Species				
*Porphyromonas pasteri*	2.51	0.92	3.98	0.048
*Haemophilus parahaemolyticus* group	1.37	0.08	3.74	0.012
*Prevotella nanceiensis* group	1.01	0.24	3.57	0.038
*Gemella haemolysans* group	0.55	0.24	3.28	0.021

LEfSe analysis included all taxa, including taxa with proportions < 1%. Taxa with an LDA effect size value > 3 were described

Hotelling’s t-test was used to compare bacterial profiles among categories

*LEfSe* Linear discriminant analysis effect size, *LDA* Linear discriminant analysis

^¶^Statistical significance was set at *p* < 0.05

We additionally conducted a LEfSe analysis to identify the most relevant functional pathways responsible for the differences between the stable and treatment groups. Among the KEGG pathways, histidine metabolism was significantly abundant in the treatment group (*p* = 0.029, LDA effect size = 2.14). Some inter-group differences were also observed in the KEGG orthologs; 13 were more abundant in the treatment group, while 6 were more abundant in the stable group (Supplementary Fig. S2 and Table S1).

For the stable group, who were observed without antibiotic treatment, the microbiomes were compared in 21 baseline sputum samples and 13 follow-up samples; the beta-diversity analysis using generalized UniFrac did not show significant differences in taxa distribution between these two sets of specimens. Furthermore, the LEfSe analysis did not identify taxa with significant abundance exceeding an LDA effect size of > 3 (Supplementary Fig. S3).

### Comparison of taxa between patients who achieved or did not achieve spontaneous culture conversion in the stable group

In the stable group, we compared the baseline sputum microbiomes of nine patients who achieved spontaneous culture conversion without antibiotics with those of 12 patients who did not achieve conversion (Table 3). At the genus level, *Bergeyella* was significantly less common in the patients who achieved spontaneous culture conversion compared to those with persistent positive NTM cultures (LDA effect size = 3.04, *p* = 0.039). At the species level, *Prevotella oris* was less common in patients who achieved spontaneous culture conversion (LDA effect size = 3.64, *p* = 0.033) (Supplementary Fig. S4).

**Table 3:** Comparison of bacterial taxa between patients who achieved or did not achieve spontaneous culture conversion in the stable group using LEfSe analysis

**Taxon name**	**Spontaneous culture conversion (%) (*n* = 9)**	**Persistent culture positive (%) (*n* = 12)**	**LDA effect size**	***P*-value**^¶^
Genus				
*Bergeyella*	0.11	0.32	3.04	0.039
Species				
*Prevotella oris*	0.39	1.07	3.64	0.033

LEfSe analysis included all taxa, including taxa with proportions < 1%. Taxa with an LDA effect size value > 3 were described

Hotelling’s t-test was used to compare bacterial profiles among categories

*LEfSe* Linear discriminant analysis effect size, *LDA* Linear discriminant analysis

^¶^Statistical significance was set at *p* < 0.05

To compare the level of microbial diversity between the two subgroups, we performed alpha-diversity analysis, and no significant differences were found (ACE, Chao1, Jackknife, and number of operational taxonomic units, all *p* > 0.05). Beta-diversity analysis using the generalized UniFrac method was performed to assess the microbial distance between the two subgroups. However, there were no significant differences at either the genus or species level (all *p* > 0.05).

### Comparison of taxa between patients who achieved or did not achieve treatment success in the treatment group

We compared the sputum microbiome at the time of antibiotic initiation between seven patients who achieved treatment success after starting antibiotic therapy and seven patients who were treatment-refractory in the treatment group (Table 4). At the genus level, in the treatment success subgroup, *Haemophilus* and *Rothia* were significantly more common (LDA effect size = 4.70 and 4.09, respectively; both *p* < 0.05), while in the treatment-refractory subgroup, *Atopobium* and *Parvimonas* were more common (LDA effect size = 4.02 and 3.67, respectively; both *p* < 0.05). In addition, at the species level, *H. parainfluenzae* was more common in patients with treatment success than in treatment-refractory patients (LDA effect size = 4.74, *p* = 0.013) (Supplementary Fig. S5).

**Table 4:** Comparison of bacterial taxa between patients who achieved or did not achieve treatment success in the treatment group using LEfSe analysis

**Taxon name**	**Treatment success (%) (*n* = 7)**	**Treatment-refractory (%) (*n* = 7)**	**LDA effect size**	***P*-value**^¶^
Genus				
*Haemophilus*	14.36	4.14	4.70	0.025
*Rothia*	3.80	1.61	4.09	0.025
*Atopobium*	0.34	2.30	4.02	0.029
*Parvimonas*	0.09	0.53	3.67	0.042
Species				
*Haemophilus parainfluenzae* group	13.99	2.93	4.74	0.013

LEfSe analysis included all taxa, including taxa with proportions < 1%. Taxa with an LDA effect size value > 3 were described

Hotelling’s t-test was used to compare bacterial profiles among categories

*LEfSe* Linear discriminant analysis effect size, *LDA* Linear discriminant analysis

^¶^Statistical significance was set at *p* < 0.05

In the alpha-diversity analysis of both subgroups, the treatment-refractory subgroup tended to have relatively greater species richness (Jackknife, Wilcoxon rank-sum test, *p* = 0.035) (Supplementary Fig. S6). Beta-diversity analysis using the generalized UniFrac method to compare the two subgroups did not show a significant difference at either the genus or species level.

## Discussion

In this study, we demonstrated the presence of distinct bacterial taxa in the sputum of stable NTM-PD patients, including species such as *Porphyromonas pasteri*, *H. parahaemolyticus*, *Prevotella nanceiensis*, and *G. haemolysans*, in comparison to patients requiring antibiotic therapy. We also identified several taxa that were relatively increased in patients with a favorable response to antibiotic therapy or decreased in patients that achieved spontaneous culture conversion. These results suggest that the microbial environment may help maintain disease stability despite NTM infection or assist in the control of NTM in patients with NTM-PD. We also believe that our data have potential for predicting the clinical course of NTM-PD through an analysis of the respiratory microbial environment.

One of our most notable findings was that, in the sputum of NTM-PD patients who remained stable without deterioration, several species belonging to the normal bacterial flora were more predominant. As of yet, the roles of the abovementioned species in the pathophysiology of NTM-PD have not been elucidated, although they have been mentioned in other microbiome studies. Anaerobic *Porphyromonas* species are commonly found in healthy individuals [15]. However, a study reported reduced lung function and the presence of *Porphyromonas pasteri* in cystic fibrosis patients [16]. Japanese researchers noted a significant presence of anaerobic bacteria in the bronchoalveolar lavage fluid of NTM-PD patients, possibly resulting from the consumption of oxygen by NTM [17]. Moreover, a recent study revealed a higher prevalence of *P. pasteri* in the oral cavity of healthy individuals compared to those with oral squamous cell carcinoma [18]. Nevertheless, the exact relevance of *Porphyromonas* to the clinical course of NTM-PD remains uncertain. Additionally, *Haemophilus* and *Prevotella* coexist in the lower respiratory tract of healthy individuals at relatively low densities. Some *Haemophilus* species can maintain the respiratory microbiome balance by competing with harmful bacteria [19]. However, the precise connection between the respiratory microbiome and *Haemophilus* remains unclear. Furthermore, recent in vivo research has shown that *Prevotella nanceiensis* alleviates neutrophilic airway inflammation and reduces inflammatory cytokines [20]. In another recent study, *G. haemolysans* was found to be significantly reduced in the oral cavity of COVID-19 patients [21]. These findings suggest that disease progression in NTM-PD may be delayed in a microbiota environment where respiratory commensal bacteria are maintained. However, this hypothesis must be verified through additional analyses.

In our study, the functional profiles related to histidine metabolism in microbes appeared to be more dominant in the treatment group. Histidine biosynthesis in bacteria plays a vital role in their survival by providing them with an essential amino acid necessary for various cellular processes. A recent study reported that histidine biosynthesis is crucial for the survival of *Mycobacterium tuberculosis*, and de novo histidine biosynthesis helps the pathogen evade host immune mechanisms [22]. Considering these findings, it can be speculated that the increase in histidine biosynthesis in the treatment group, compared to the stable group in our study, suggests a relevance to the progression of NTM-PD. However, with the current data alone, it is difficult to establish causality between disease status and microbial distribution. Further research is needed.

In patients with stable NTM-PD, we observed no significant differences in alpha- or beta-diversity between the baseline and follow-up sputum samples. Furthermore, LEfSe analysis did not identify any taxa with significant differences between these two sets of samples. Given that these patients showed no clinical or radiological deterioration during their follow-up, our findings provide additional support for the concept of a particular microbial environment associated with stability in NTM-PD patients. Conceivably, the surrounding microbial community may continuously stimulate an appropriate immune response in the host [23], or certain microbial communities might inhibit NTM proliferation via bacterial antagonism. Therefore, evaluating diversity and microbial distribution during follow-up could be valuable for predicting the clinical course of NTM-PD. Nevertheless, our study alone is insufficient to establish a causal relationship, so further research is needed.

In our study, within the treatment group, the genera *Haemophilus* and *Rothia*, and the *H. parainfluenzae* group, were more abundant in the sputum of patients who achieved treatment success compared to those who were treatment-refractory. Both *Rothia* and *H. parainfluenzae* are normal oral or respiratory commensals [24–27]. A recent study showed that *R. mucilaginosa* acts as an anti-inflammatory bacterium in the respiratory tract of patients with chronic lung disease [28]; the study demonstrated that *R. mucilaginosa* inhibits pathogen-associated pro-inflammatory responses in a mouse model, and the abundance of *Rothia* species was negatively correlated with pro-inflammatory markers such as interleukin-8, IL-1β, and matrix metalloproteinase in the sputum of bronchiectasis patients. In addition, a recent Chinese study found that *H. parainfluenzae* was more predominant in the bronchoalveolar lavage fluid of patients with negative tuberculosis cultures than in those with positive cultures [29]. Thus, our data suggest that a microbial environment closer to the normal flora may predict more favorable treatment outcomes in NTM-PD. However, more research is required to investigate the interaction between NTM and the microbiome. Additionally, in our study, *Atopobium* and *Parvimonas* exhibited relatively higher distributions in the treatment-refractory group compared to the treatment success group. In a previous study that analyzed bronchoalveolar lavage fluid, including 71 sarcoidosis patients, *Atopobium* was reported to be more abundant compared to controls [30]. *Parvimonas* was also found to have a higher distribution in the sputum of tuberculosis patients compared to health controls [31]. These findings suggest that specific bacterial taxa may be associated with chronic inflammatory responses or mycobacterial infections; however, their biological function or interaction with host immunity has yet to be further elucidated.

In the stable group, the genus *Bergeyella* and *Prevotella oris* were decreased in the sputum of patients with spontaneous culture conversion. This finding is consistent with observations in studies that included lung cancer and cystic fibrosis patients, where the prevalence of *Bergeyella* and *P. oris* in respiratory samples of patients with these diseases was higher than in controls [32, 33]. However, further research is needed to understand the roles of these taxa in NTM-PD patients.

Notably, the sputum samples at the initiation of treatment for the treatment-refractory patients in this study had greater species richness in the alpha-diversity analysis compared to the successfully treated patients. This result contrasts the common finding in other respiratory diseases whereby higher diversity is indicative of a better prognosis. Increased diversity is likely associated with more severe bacterial invasion, and this observation aligns with a recent study of lung tissues of NTM-PD patients, where areas of more severe lung parenchymal damage had greater bacterial diversity [11].

Our study had several limitations. First, the number of patients included was small. Nonetheless, we believe our paper still holds research value because studies analyzing respiratory samples from NTM-PD patients according to clinical outcomes are rare. Due to the long clinical course and treatment periods in NTM-PD patients, continuously collecting well-selected respiratory samples is challenging. Based on the small-scale study, our research team is planning further studies to analyze microbiome changes according to significant clinical events in NTM-PD patients. Second, 16S rRNA gene sequencing has limitations for identifying bacteria, so there might be unidentified taxa that were missed using this technique. Third, most of our subjects were females who had the nodular bronchiectatic radiological phenotype of NTM-PD, which could have influenced the respiratory microbiomes. Another limitation in our study is the coexistence of rapidly growing mycobacteria (such as *M. abscessus* or *M. massiliense*) or mixed etiologies alongside the *M. avium* complex as causative agents. Therefore, these factors may have influenced our study results. However, when comparing the *M. avium* complex with other species categories, there were no significant differences between the stable and treatment groups. Given that it is not yet known whether the microbial environment varies specifically depending on the causative NTM species, further studies are needed. In addition, our study does not establish causality. Therefore, to address this limitation, it is necessary to collect longitudinal data over an extended period, starting from the pre-diagnostic stage through disease diagnosis and treatment, or to attempt more detailed functional analysis. Because we did not use induced sputum, we cannot rule out the influence of salivary contamination. However, we visually confirmed sufficient specimens during sputum collection and used only specimens where NTM was cultured from the samples collected for microbiome research. Lastly, in the LEfSe analysis, we presented results showing significant differences and p-values between the two patient groups in tables. As is commonly done in microbiome studies, we utilized the LEfSe technique. However, upon examining q-values to lower the false discovery rate more strictly, we found that the q-value in our data was > 0.05. We attribute this to the relatively small number of sputum samples from our patients and the large number of microbes requiring repeated comparison. Therefore, given the relatively small sample size of our study, we deemed it inappropriate to rely solely on q-values for determining significance. Instead, we increased reliability by adjusting taxa exceeding an LDA cut-off of > 3, rather than > 2. We acknowledge the influence of various environmental factors on microbial distribution, inherent in microbiome research. However, since it is impossible to completely eliminate all biases, we conducted this study analysis targeting the most carefully selected patients who had typical clinical presentations of NTM-PD.

In conclusion, we demonstrated the presence of distinct bacterial taxa in the sputum of NTM-PD patients with a stable status compared to those requiring treatment. Several taxa were relatively increased in patients with a favorable response to antibiotics and decreased in patients that achieved spontaneous culture conversion. Our findings suggest the existence of a microbial environment that may help maintain disease stability or aid in the control of NTM in patients with NTM-PD.

### Supplementary Information

**Supplementary Material 1.**

## Data Availability

The raw data were registered in the Sequence Read Archive data (Bioproject.) They are available under the accession number PRJNA1048558(Release date: 2024-03-01).

## Abbreviations

NTM: Nontuberculous mycobacteria
PD: Pulmonary disease
IRB: Institutional review board
MPT: Microbial taxonomic profiling
LDA: Linear discriminant analysis
LEfSe: Linear discriminant analysis effect size

## References

[1] Prevots DR, Marras TK (2015). Epidemiology of human pulmonary infection with nontuberculous mycobacteria: a review. Clin Chest Med.

[2] Griffith DE, Aksamit T, Brown-Elliott BA, Catanzaro A, Daley C, Gordin F (2007). An official ATS/IDSA statement: diagnosis, treatment, and prevention of nontuberculous mycobacterial diseases. Am J Respir Crit Care Med.

[3] Daley CL, Iaccarino JM, Lange C, Cambau E, Wallace RJ, Andrejak C (2020). Treatment of nontuberculous mycobacterial pulmonary disease: an official ATS/ERS/ESCMID/IDSA clinical practice guideline. Eur Respir J.

[4] Kartalija M, Ovrutsky AR, Bryan CL, Pott GB, Fantuzzi G, Thomas J (2013). Patients with nontuberculous mycobacterial lung disease exhibit unique body and immune phenotypes. Am J Respir Crit Care Med.

[5] Hwang JA, Kim S, Jo KW, Shim TS (2017). Natural history of *Mycobacterium avium c*omplex lung disease in untreated patients with stable course. Eur Respir J.

[6] Kim SJ, Park J, Lee H, Lee YJ, Park JS, Cho YJ (2014). Risk factors for deterioration of nodular bronchiectatic *Mycobacterium avium* complex lung disease. Int J Tuberc Lung Dis.

[7] Pan SW, Shu CC, Feng JY, Wang JY, Chan YJ, Yu CJ (2017). Microbiological persistence in patients with Mycobacterium avium complex lung disease: the predictors and the impact on radiographic progression. Clin Infect Dis.

[8] Kwon BS, Lee JH, Koh Y, Kim WS, Song JW, Oh YM (2019). The natural history of non-cavitary nodular bronchiectatic *Mycobacterium avium* complex lung disease. Respir Med.

[9] Cuthbertson L, Walker AW, Oliver AE, Rogers GB, Rivett DW, Hampton TH (2020). Lung function and microbiota diversity in cystic fibrosis. Microbiome.

[10] O’Dwyer DN, Dickson RP, Moore BB (2016). The lung microbiome, immunity, and the pathogenesis of chronic lung disease. J Immunol.

[11] Kim BG, Kang N, Kim SY, Kim DH, Kim H, Kwon OJ (2023). The lung microbiota in nontuberculous mycobacterial pulmonary disease. PLoS One.

[12] Kim BG, Yu JY, Kim SY, Kim DH, Jhun BW (2023). Changes in sputum microbiota during treatment for nontuberculous mycobacterial pulmonary disease. Sci Rep.

[13] Jhun BW, Kim SY, Moon SM, Jeon K, Kwon OJ, Huh HJ (2018). Development of macrolide resistance and reinfection in refractory *Mycobacterium avium* complex lung disease. Am J Respir Crit Care Med.

[14] Tango CN, Seo SS, Kwon M, Lee DO, Chang HK, Kim MK (2020). Taxonomic and functional differences in cervical microbiome associated with cervical cancer development. Sci Rep.

[15] Guilloux CA, Lamoureux C, Beauruelle C, Héry-Arnaud G (2021). Porphyromonas: a neglected potential key genus in human microbiomes. Anaerobe.

[16] Webb K, Zain NMM, Stewart I, Fogarty A, Nash EF, Whitehouse JL (2022). *Porphyromonas pasteri* and *Prevotella nanceiensis* in the sputum microbiota are associated with increased decline in lung function in individuals with cystic fibrosis. J Med Microbiol.

[17] Yamasaki K, Mukae H, Kawanami T, Fukuda K, Noguchi S, Akata K (2015). Possible role of anaerobes in the pathogenesis of nontuberculous mycobacterial infection. Respirology.

[18] Yang CY, Yeh YM, Yu HY, Chin CY, Hsu CW, Liu H (2018). Oral microbiota community dynamics associated with oral squamous cell carcinoma staging. Front Microbiol.

[19] Latham RD, Gell DA, Fairbairn RL, Lyons AB, Shukla SD, Cho KY (2017). An isolate of *Haemophilus haemolyticus* produces a bacteriocin-like substance that inhibits the growth of nontypeable *Haemophilus influenzae*. Int J Antimicrob Agents.

[20] Larsen JM, Musavian HS, Butt TM, Ingvorsen C, Thysen AH, Brix S (2015). Chronic obstructive pulmonary disease and asthma-associated *Proteobacteria*, but not commensal *Prevotella* spp., promote Toll-like receptor 2-independent lung inflammation and pathology. Immunology.

[21] Liu J, Liu S, Zhang Z, Lee X, Wu W, Huang Z (2021). Association between the nasopharyngeal microbiome and metabolome in patients with COVID-19. Synth Syst Biotechnol.

[22] Dwivedy A, Ashraf A, Jha B, Kumar D, Agarwal N, Biswal BK (2021). De novo histidine biosynthesis protects *Mycobacterium tuberculosis* from host IFN-γ mediated histidine starvation. Commun Biol.

[23] Zheng D, Liwinski T, Elinav E (2020). Interaction between microbiota and immunity in health and disease. Cell Res.

[24] Natalini JG, Singh S, Segal LN (2023). The dynamic lung microbiome in health and disease. Nat Rev Microbiol.

[25] Segal LN, Clemente JC, Tsay JC, Koralov SB, Keller BC, Wu BG (2016). Enrichment of the lung microbiome with oral taxa is associated with lung inflammation of a Th17 phenotype. Nat Microbiol.

[26] Liljemark WF, Bloomquist CG, Uhl LA, Schaffer EM, Wolff LF, Pihlstrom BL (1984). Distribution of oral *Haemophilus* species in dental plaque from a large adult population. Infect Immun.

[27] Biesbroek G, Tsivtsivadze E, Sanders EA, Montijn R, Veenhoven RH, Keijser BJ (2014). Early respiratory microbiota composition determines bacterial succession patterns and respiratory health in children. Am J Respir Crit Care Med.

[28] Rigauts C, Aizawa J, Taylor SL, Rogers GB, Govaerts M, Cos P (2022). *Rothia mucilaginosa* is an anti-inflammatory bacterium in the respiratory tract of patients with chronic lung disease. Eur Respir J.

[29] Ding L, Liu Y, Wu X, Wu M, Luo X, Ouyang H (2021). Pathogen metagenomics reveals distinct lung microbiota signatures between bacteriologically confirmed and negative tuberculosis patients. Front Cell Infect Microbiol.

[30] Zimmermann A, Knecht H, Häsler R, Zissel G, Gaede KI, Hofmann S (2017). *Atopobium* and *Fusobacterium* as novel candidates for sarcoidosis-associated microbiota. Eur Respir J.

[31] Cheung MK, Lam WY, Fung WY, Law PT, Au CH, Nong W (2013). Sputum microbiota in tuberculosis as revealed by 16S rRNA pyrosequencing. PLoS One.

[32] Druzhinin VG, Matskova LV, Demenkov PS, Baranova ED, Volobaev VP, Minina VI (2020). Taxonomic diversity of sputum microbiome in lung cancer patients and its relationship with chromosomal aberrations in blood lymphocytes. Sci Rep.

[33] O’Connor JB, Mottlowitz MM, Wagner BD, Boyne KL, Stevens MJ, Robertson CE (2021). Divergence of bacterial communities in the lower airways of CF patients in early childhood. PLoS One.

