# Supplementary material for: Comparison of the sputum microbiome between patients with stable nontuberculous mycobacterial pulmonary disease and patients requiring treatment
Source: BMC Microbiol. 2024 May 18;24:172. doi: 10.1186/s12866-024-03308-2 (PMC11102115; doi:10.1186/s12866-024-03308-2)

Supplementary material

Supplementary Figure S1. Comparison of abundance differences at the bacterial genus or species level between the baseline sputum of the stable group (n = 21) and the sputum of the treatment group (initiation of antibiotics group; n = 14).

(A) *Gemella* (Wilcoxon rank-sum test, p = 0.017)

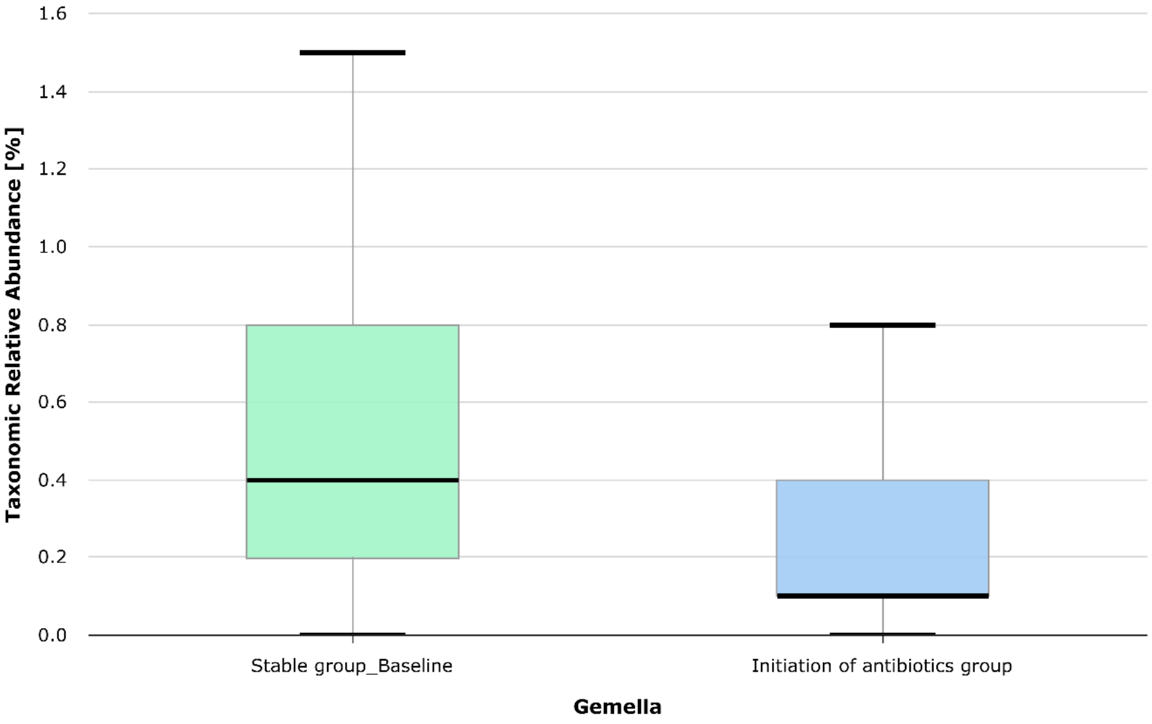

(B) *Porphyromonas pasteri* (Wilcoxon rank-sum test, p = 0.053)

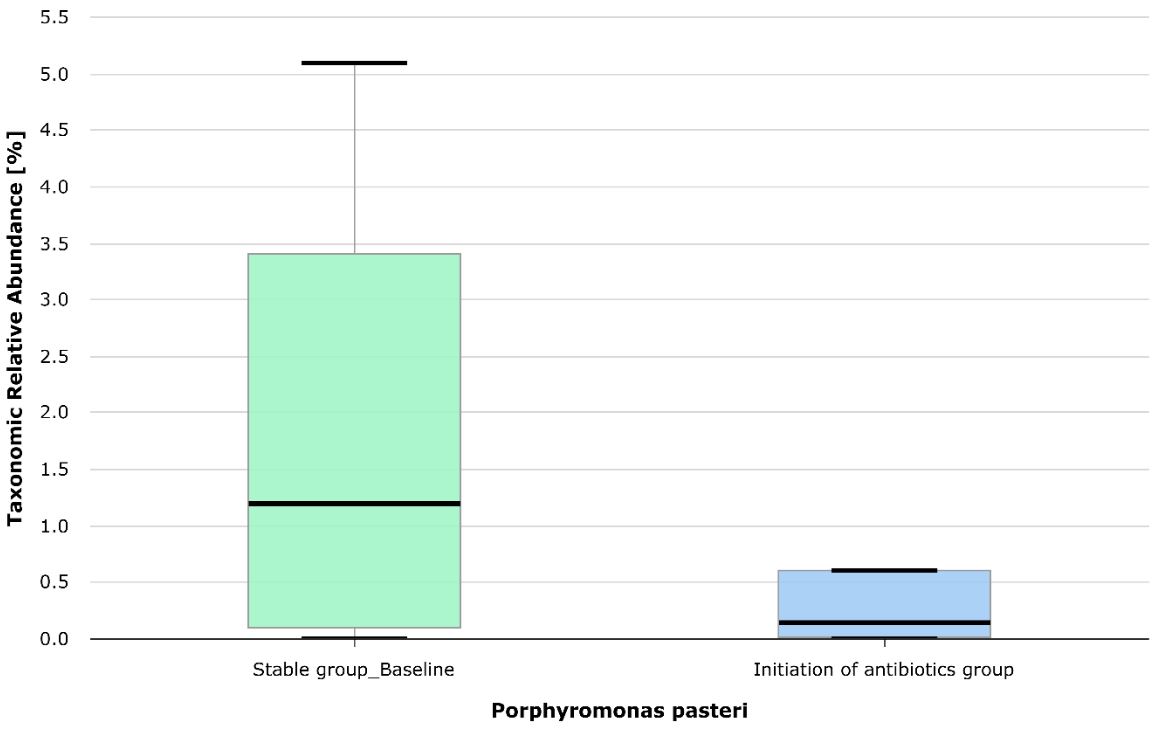

(C) *Haemophilus parahaemolyticus* (Wilcoxon rank-sum test, p = 0.024)

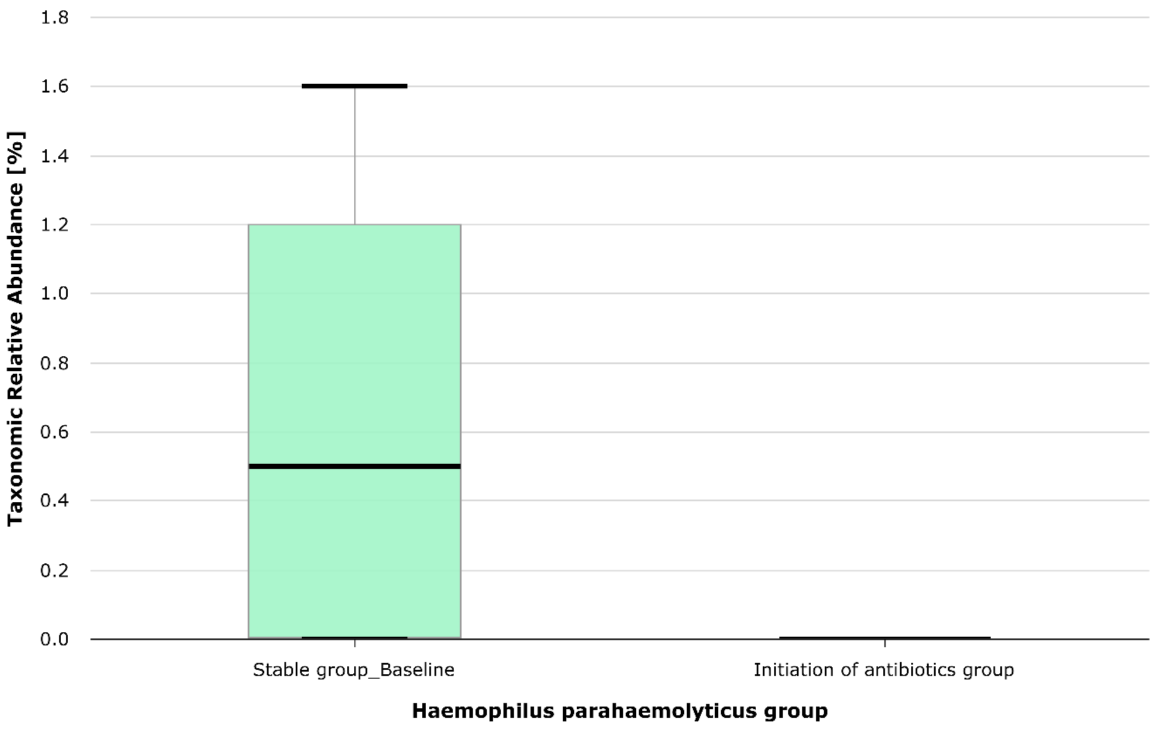

(D) *Prevotella nanceiensis* (Wilcoxon rank-sum test, p = 0.045)

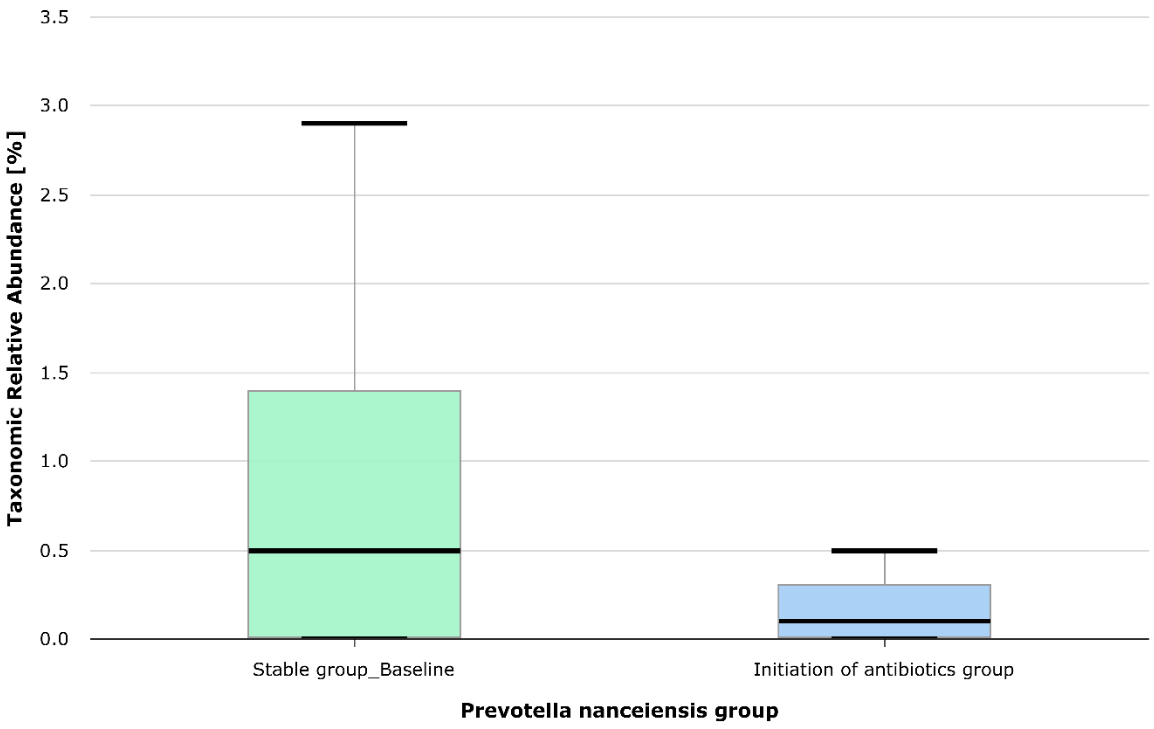

(E) *Gemella haemolysans* (Wilcoxon rank-sum test, p = 0.022)

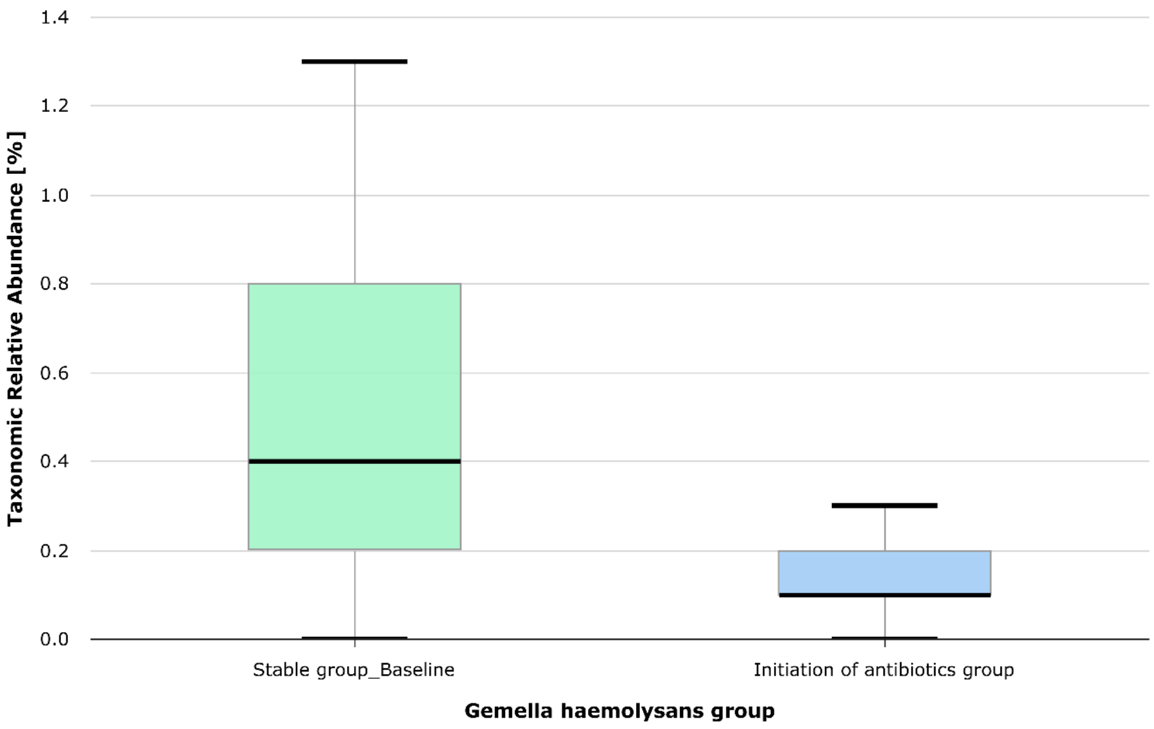

Supplementary Figure S2. Difference of in Kyoto Encyclopedia of Genes and Genomes (KEGG) pathway profiles between groups by LEfSe analysis (Logarithmic LDA score >1.5; p-value <0.05).

(A) Pathway enrichment for KEGG cellular processes in treatment groups.

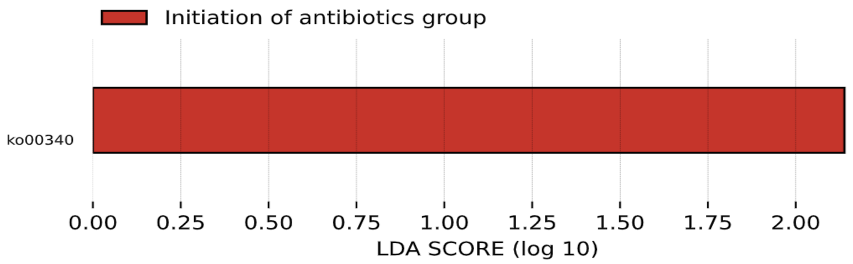

(B) Orthology enrichment for KEGG cellular processes in stable group and treatment group.

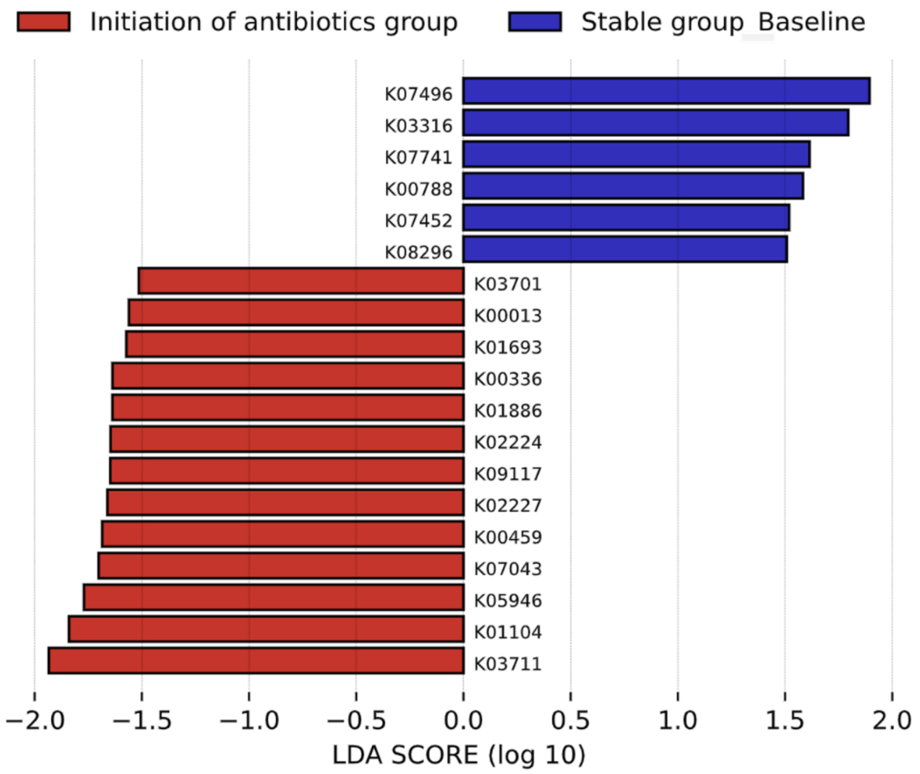

Supplementary Table S1. Mean relative abundances of 1 KEGG pathways and 2 KEGG orthologs involved in pathways identified using LEfSe

KEGG Pathway	Pathway Name	Stable group	Treatment group	p-value	Orthology	Definition	Stable group	Treatment group	p-value
Ko00340	Histidine metabolism	0.25123	0.28278	0.0286	K01693	Imidazoleglycerol-phosphate dehydratase	0.02385	0.03162	0.01271
					K00013	Histidinol dehydrogenase	0.03219	0.03921	0.02016

Analysis at the p-value of 0.05.

Supplementary Figure S3. Beta-diversity between baseline sputum (n = 21) and follow-up sputum (n = 13) in the stable group. Beta-diversity analysis using Generalized UniFrac did not show significant differences in bacterial taxa distribution between the two specimen groups. Furthermore, the LEfSe analysis did not identify taxa with significant abundance exceeding an LDA effect size of > 3.

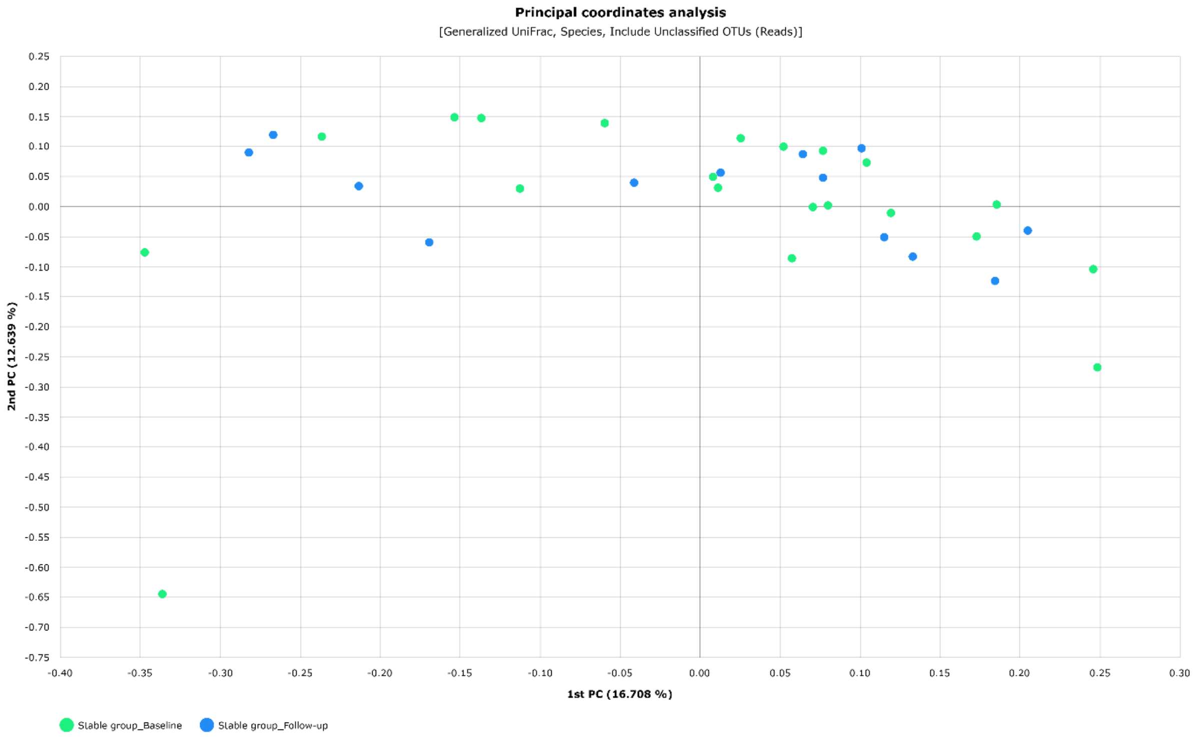

Supplementary Figure S4. Comparison of abundance differences in bacterial taxa between the baseline sputum of patients with spontaneous culture conversion (n = 9) and patients with persistent culture positivity (n = 12) in the stable group.

(A) *Bergeyella* (Wilcoxon rank-sum test, p = 0.043)

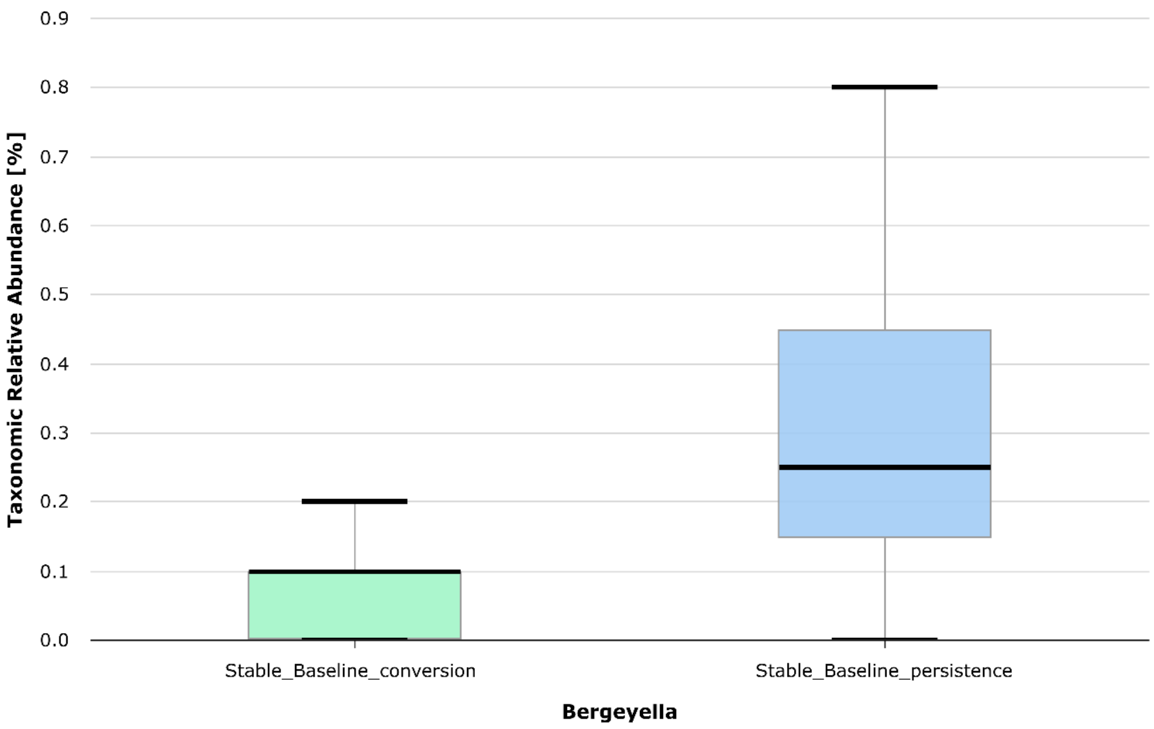

(B) *Prevotella oris* (Wilcoxon rank-sum test, p = 0.036)

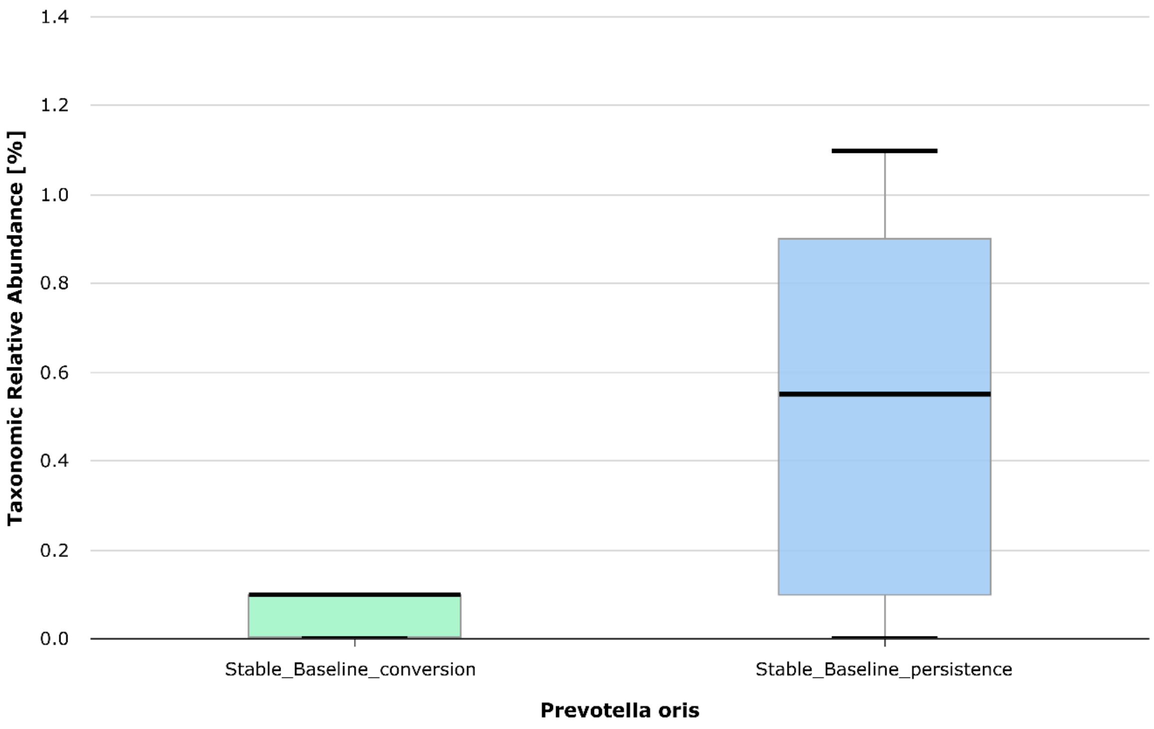

1.4
1.2
1.0
0.8
0.6
0.4
0.2
0.0
Taxonomic Relative Abundance [%]
Stable_Baseline_conversion
Stable_Baseline_persistence
Prevotella oris

Supplementary Figure S5. Comparison of abundance differences in bacterial taxa between the sputum of treatment success (n = 7) and treatment-refractory (n = 7) patients in the treatment group.

(A) *Haemophilus* (Wilcoxon rank-sum test, p = 0.025)

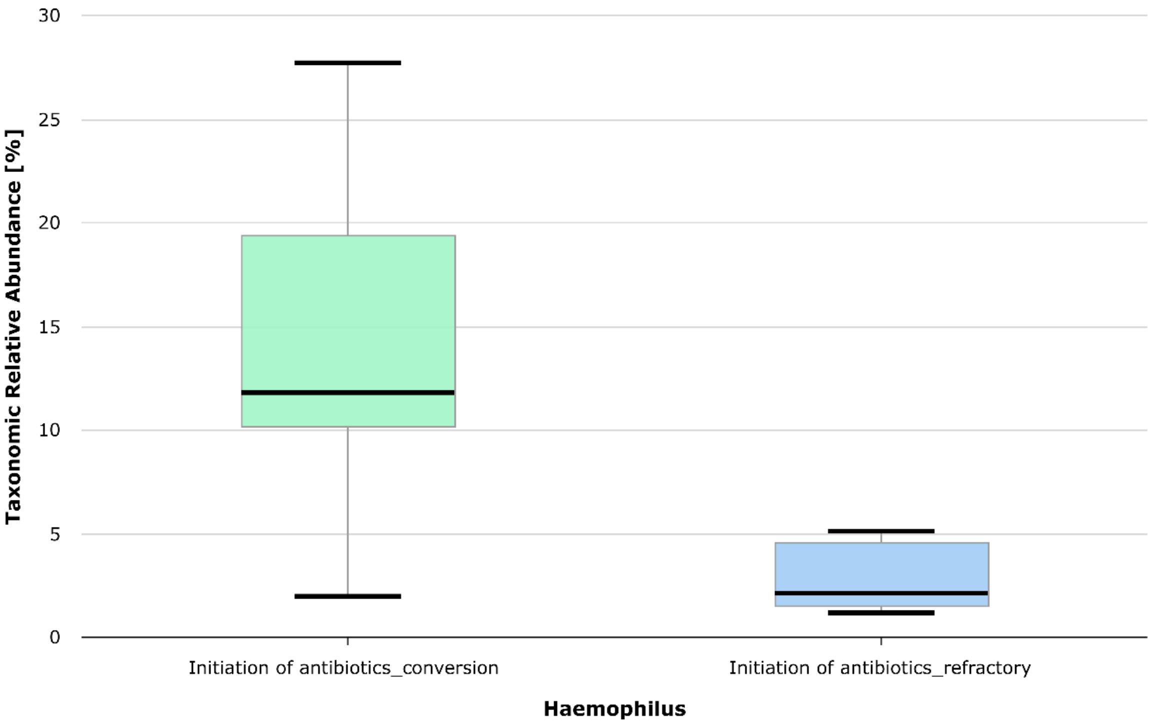

(B) *Rothia* (Wilcoxon rank-sum test, p = 0.025)

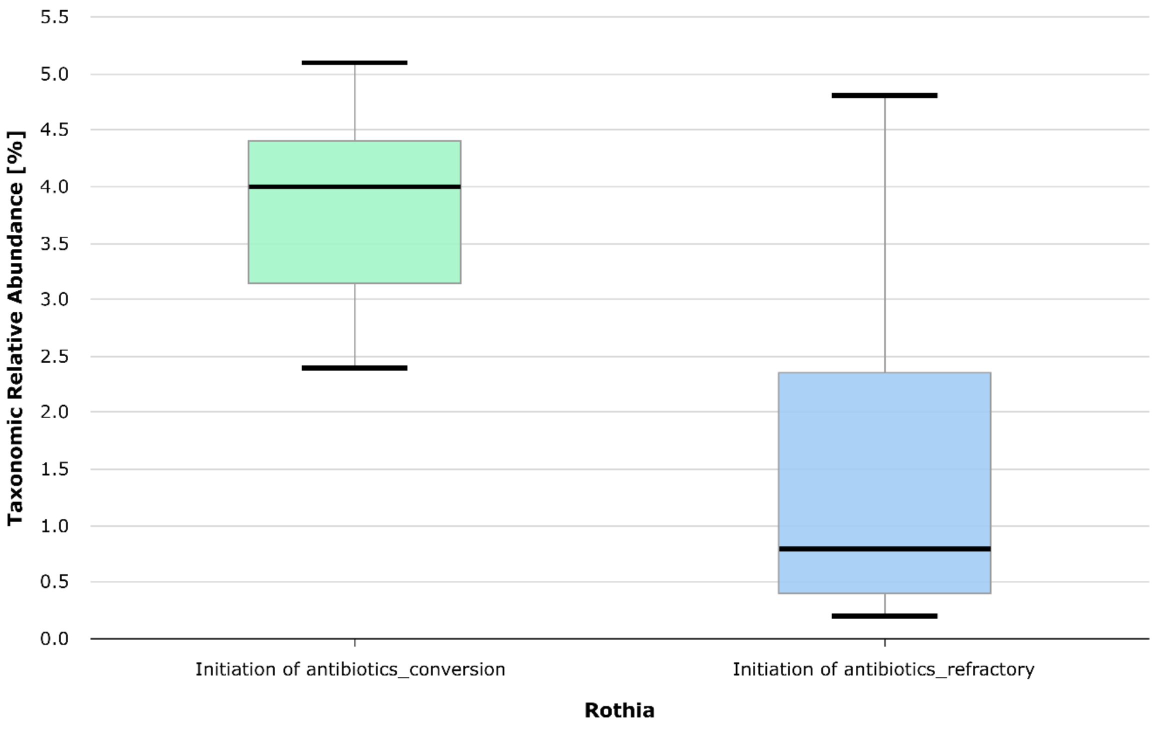

(C) *Atopobium* (Wilcoxon rank-sum test, p = 0.030)

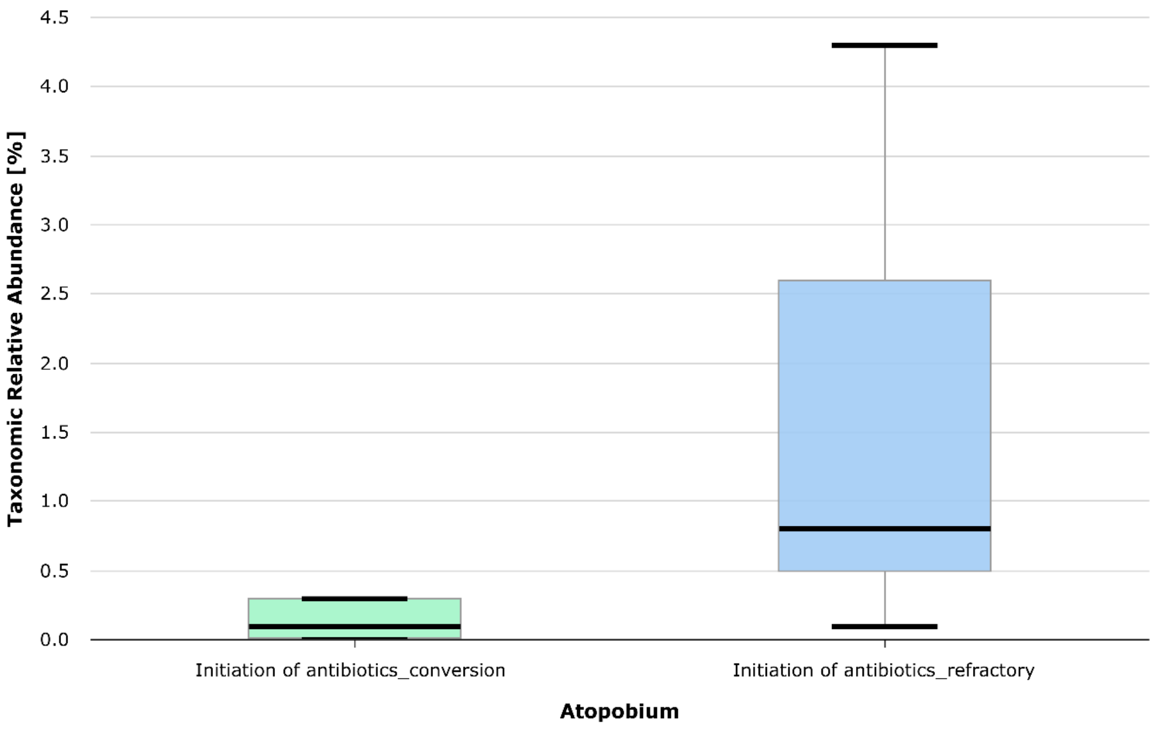

(D) *Parvimonas* (Wilcoxon rank-sum test, p = 0.048)

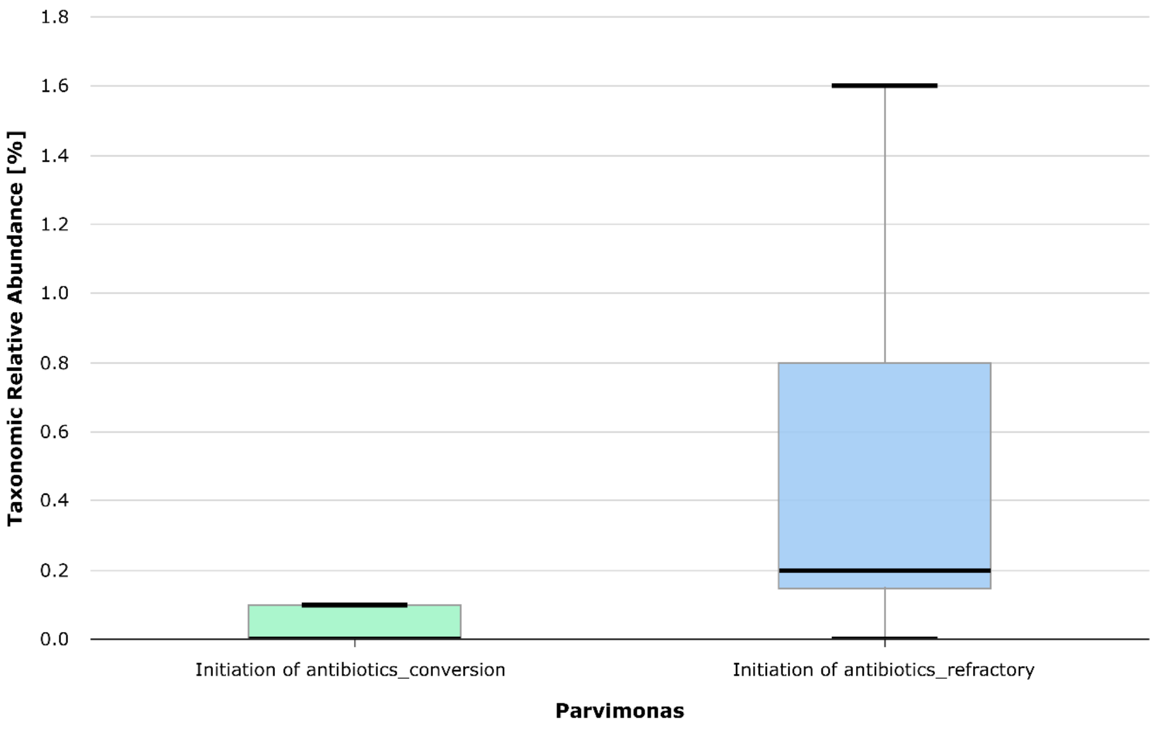

(E) *Haemophilus parainfluenzae* group (Wilcoxon rank-sum test, p = 0.013)

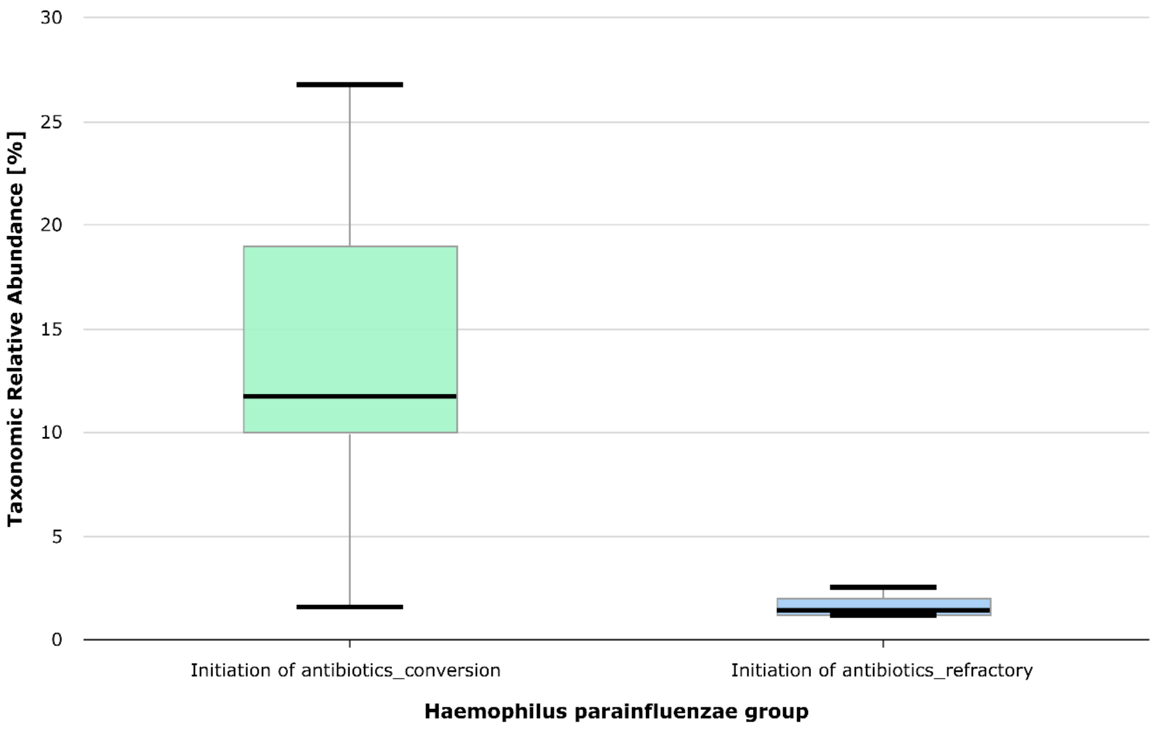

Supplementary Figure S6. Difference in bacterial species richness of sputum at treatment initiation between the treatment-success patients (n = 7) and the treatment-refractory patients (n = 7) (Jackknife, Wilcoxon rank-sum test, p = 0.035).

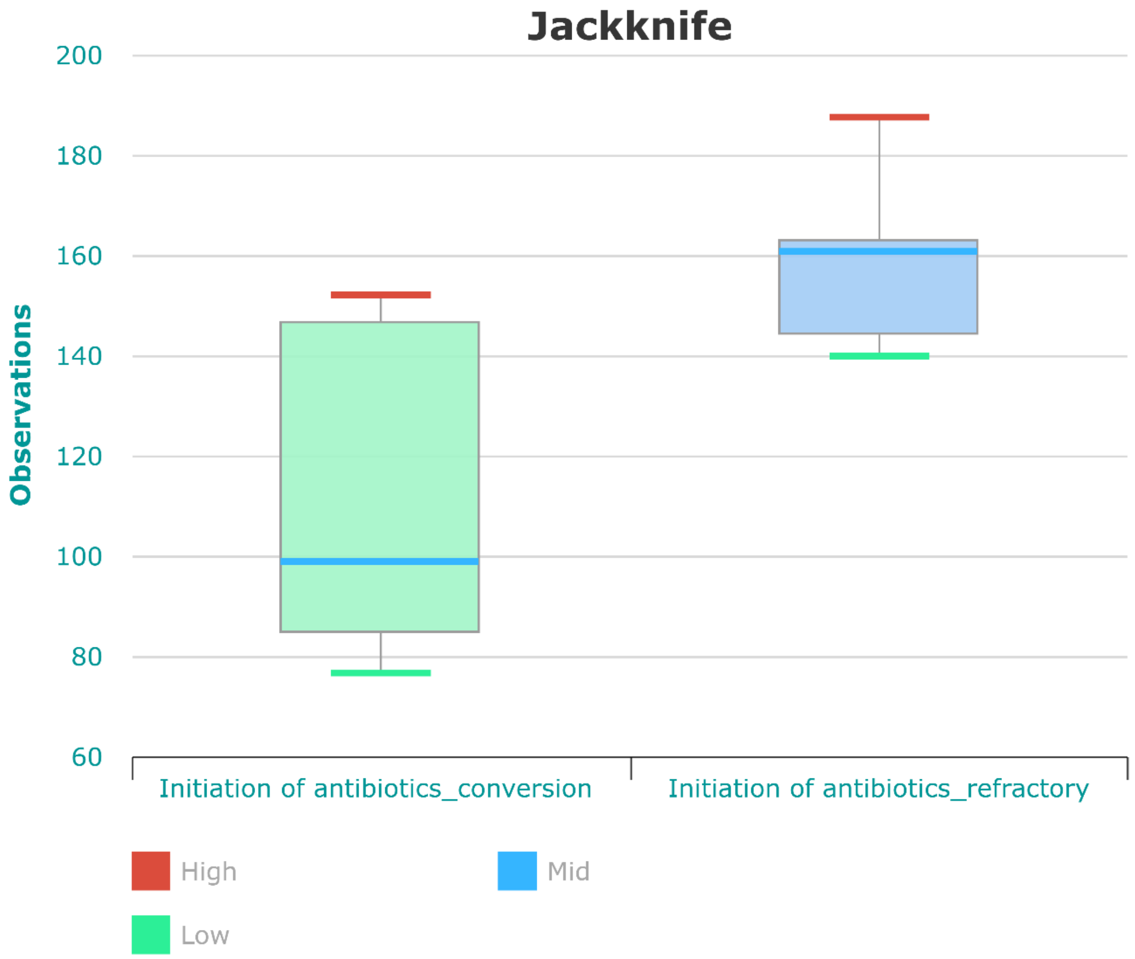

Supplement: Supplementary file 1 — Supplementary Material 1. [file 12866_2024_3308_MOESM1_ESM.pdf]
